# Transversal incision of the vagina favors the remaining of the tape in the middle-third urethra compared to longitudinal incision during transobturator sling procedures for stress urinary incontinence

**DOI:** 10.1186/s12893-015-0071-8

**Published:** 2015-07-17

**Authors:** L. Pirtea, I. Sas, Razvan Ilina, D. Grigoraș, O. Mazilu

**Affiliations:** Department of Obstetrics and Gynecology, University of Medicine and Pharmacy “Victor Babeş”, Piața Eftimie Murgu, nr. 2, Timișoara, Romania; Department of Surgery, University of Medicine and Pharmacy “Victor Babeş”, Str. Dimitrie Cantemir, nr. 1, Timişoara, 300001 Romania

**Keywords:** Transobturator sling, Urinary incontinence, Transversal incision, Mid-urethra, de novo urgency

## Abstract

**Background:**

To describe a new type of incision of the vagina during transobturator sling procedure and to evaluate by ultrasound the tape position at 3, 6 and 12 months after surgery.

We conducted a prospective study including 51 patients with urinary stress incontinence who underwent sling procedure using the transversal vaginal incision. Tape position was evaluated by ultrasound at 3, 6 and 12 months after surgery and expressed as a percentage of the urethral length (the proximal third of the urethral length 0–39 %, the middle third 40–60 %, and the distal third 60–100 %).

Informed consent was obtained from all patients prior to their inclusion in the study. All procedures have been performed in accordance with the ethical standards laid down in the 1964 Declaration of Helsinki and its later amendments and were approved by the Institutional Review Board and Ethical Committee of “Victor Babeş” University of Medicine and Pharmacy Timisoara before the beginning of the study (no 7/17.04.2012).

**Results:**

At 3 months after surgery, 3.92 % of the slings were located in the proximal third of the urethra, 88.23 % in the middle third of the urethra and 7.84 % in the distal third. At 6 and 12 months after surgery we obtained similar results: 9.81 % of the slings were located in the proximal third of the urethra, 82.35 % in the middle third and 7.84 % in the distal third of the urethra.

**Conclusion:**

The transversal incision of the vagina offers a minimal dissection along the long axis of the urethra favoring the remaining of the tape in the middle third of the urethra.

## Background

Suburethral synthetic slings are becoming the first-line surgical method for the treatment of the urinary stress incontinence [1]. The surgical outcome of the TOT (transobturator tape) sling depends on the complex interaction between the tape and the urethra [2]. Tape position along the urethra after the mid-urethral sling procedures seems to play a role in the development of the de novo urgency and voiding difficulties. Different authors indicate that tape location outside the middle third of urethra is associated with recurrent stress incontinence and increased incidence of de novo urgency [1, 3, 4]. Sling location after surgery can be evaluated by ultrasound. Despite the fact that all slings are placed initially in the mid-urethral area 45–65 % of them were found outside the middle third of the urethra at 1 year after surgery [3–5].

Our primary goal was to find solutions to increase the percentage of slings that remain in the mid-urethral area. The length of the vertical vaginal incision varies from 1.5 to 2–3 cm according to different authors [6, 7]. Since the sling width is 1 cm there is some extra space, dissected between the urethra and the vagina, that allows the sling to slide along the urethra. We considered that a transversal incision of the vagina at the level of mid-urethra reduces the dissection along the urethra and limits the possibility of the tape to slide.

Our study demonstrates that the transversal incision of the vagina actually increases the percentage of slings that remain in the middle third of the urethra after surgery compared to the original technique.

## Methods

### Technique description

**Fig. 1:**
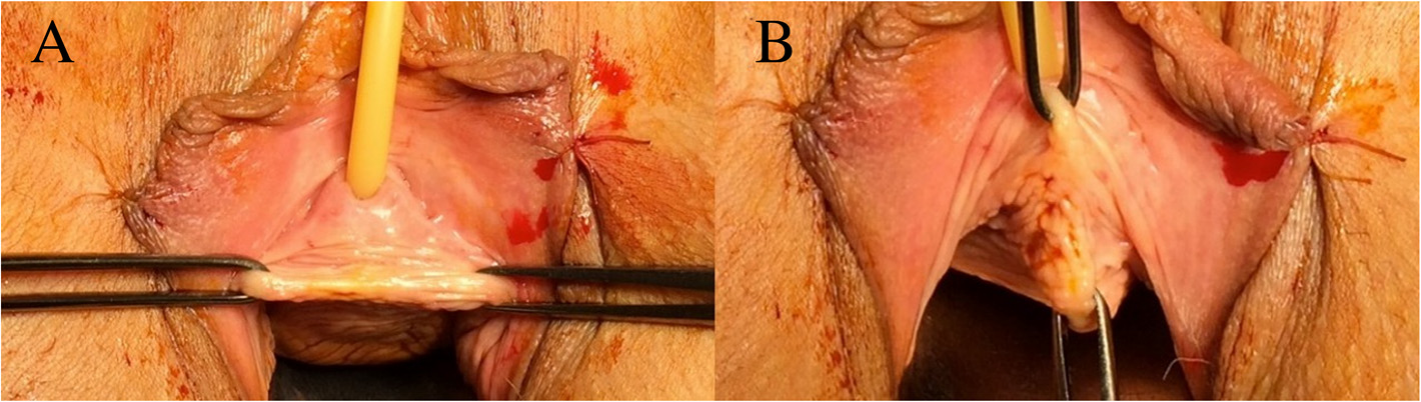
**a** Transversal line of incision at the level of mid urethra, perpendicular on the long axis of the urethra. **b** The classic line of incision

We performed TOT mid-urethral sling procedure using a transversal incision of the vagina. The incision length was 2–2.5 cm perpendicular on the long axis of the urethra. The location of the incision was at the middle of the urethral length (Fig. 1a), measured as the distance between the external uretral meatus and the bladder neck, identified by palpating the Foley catheter balloon trough the vagina. This way, the access to the peri-urethral spaces is easier and the dissection along the urethra is minimal. The classical technique uses a longitudinal incision along the long axis of the urethra (Fig. 1b). Our goal was to have minimal vaginal dissection along the long axis of the urethra.

The rest of the procedure is the same as described by Delorme: helicoidal needles are used to insert the tape under finger control via the transobturator fossa. The entry point is from the medial border of the obturator fossa at the level of the clitoris, with outside-in needle passage through the obturator fossa and placing a 1 cm wide polypropylene tape under the urethra in a tension free manner [6, 8].

### Case selection

The procedure was performed on 51 consecutive patients that were scheduled for TOT outside-in sling procedure between May 2012 and June 2013 at the Department of obstetrics gynecology of County hospital Timisoara Romania. Patients presenting urinary stress incontinence were selected. The diagnostic criterion was urine loss on cough test. Further urodynamic assessment was performed and all patients with detrusor instability were excluded. Patients with concomitant cystocele, rectocele or uterine prolapse were also excluded. No other criteria such as parity, age, body mass index were used.

**Fig. 2:**
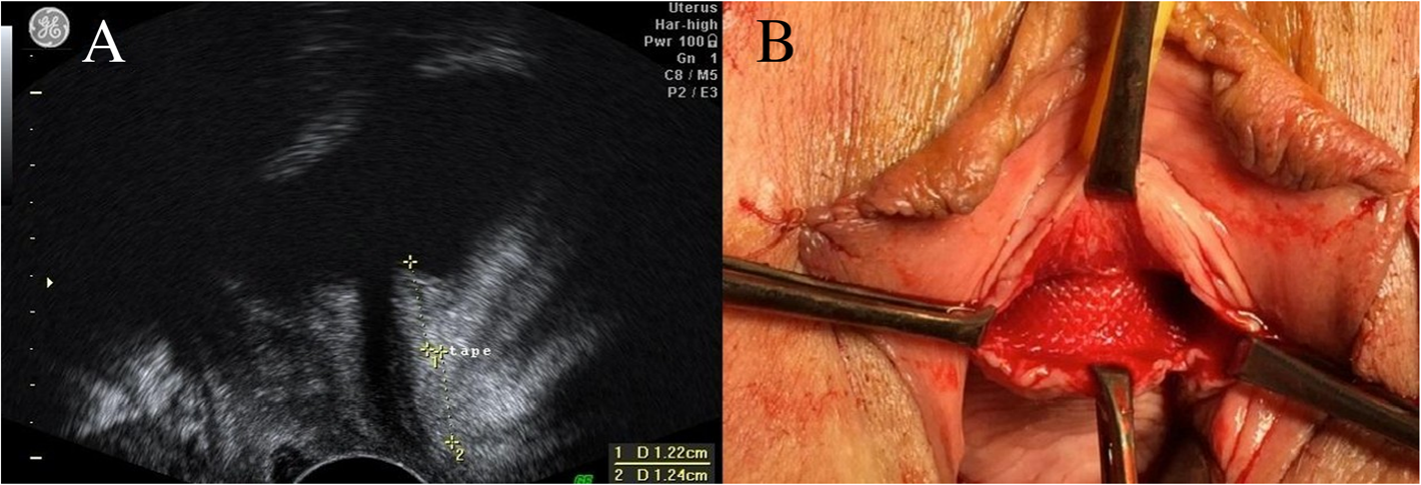
**a** Ultrasound evaluation of the tape after surgery: D1 distance from bladder neck to the middle of the tape, D2 distance from the middle of the tape to the external urethral meatus. **b** Tape under the urethra

Transobturator outside-in tension free sling procedure was performed in all cases. All procedures were performed by the same surgeon. No other surgical procedures to restore pelvic floor statics were performed. Patients were followed up at 3, 6 and 12 months after surgery. The position of the tape was evaluated by translabial ultrasound examination (Fig. 2a), performed using GE Voluson 730 PRO V (http://www3.gehealthcare.com/en/Products/Categories/Ultrasound/Voluson/Voluson_730) with transvaginal probe (4–9 MHz). All cases were evaluated by the same examinator, blinded regarding to outcome and procedure type. Patients were examined in the lithotomy position with full bladder. The distances from the bladder neck to the middle of the tape and from the bladder neck to the external urethral meatus was measured in a sagittal plane. Tape location along the urethra was expressed as a percentage of the urethral length as follows: distance from the bladder neck to the middle of the tape divided by distance from the bladder to the external urethral meatus. The proximal third of the urethra was considered the 0–39 % of the urethral length, the middle third 40–60 % of the urethral length, and the distal third 60–100 % of the urethral length. All patients completed at each follow up visit a complete urinary diary.

For sample size calculation we have used our actual value of 82.1 % success rate and as comparator the value obtained by Bogusiewicz et al. with a success rate of 54 %. We have used the one sample test of equality [9]. For α = 0.05, one sided, the required sample size for having an 80 % power (β = 0.2) for correctly detecting a difference between post treatment success rate and the reference value of 54 % is 15 patients. For the power of 90 % (β = 0.2), with all other parameters similar with above, the sample size should be 20 patients.

Our sample has 51 patients and the power of our study is over 90 %.

The study was conducted in a prospective manner. Informed consent was obtained from all patients prior to their inclusion in the study. All procedures have been performed in accordance with the ethical standards laid down in the 1964 Declaration of Helsinki and its later amendments and were approved by the Institutional Review Board and Ethical Committee of “Victor Babeş” University of Medicine and Pharmacy Timisoara before the beginning of the study (no 7/17.04.2012).

## Results

The procedure was performed in 51 patients, with a mean age of 60 years, using the same operative technique in all cases. Thirty-seven patients were postmenopausal. The mean BMI was 36 kg/m^2^. No major complications, such as major bleeding, bladder or urethral injury, were encountered. The mean operative time was 20 min and the mean blood loss was 40 ml which was calculated by using pre-weighed swabs. There was no difference in surgical time or blood loss compared to the original technique (with a longitudinal incision). The rate of continence after surgery was assessed using the cough test. The result were: 87 % patients were cured; 8.7 % significantly improved; while 4.3 % had no improvement (Table 1). There were no cases of postoperative leg or groin pain in our group. We had no cases of postoperative acute urine retention. Vaginal exposure of sling material was not encountered in any patient in our group using the modified procedure.

**Table 1:** Rate of continence after surgery

Rate of continence after surgery	
Cured	82,00 %
Significantly improved	11. 70 %
No improvement	6,30 %

On the ultrasound examination performed at 3 months after surgery the position of the tapes was as follows: 3.92 % of the slings were located in the proximal third of the urethra, 88.23 % in the middle third of the urethra and 7.84 % in the distal third. At 6 months after surgery, 9.81 % of the slings were located in the proximal third of the urethra, 82.35 % in the middle third, and 7.84 % in the distal third of the urethra. At one year the position of the tape was the same as the one observed at 6 months after surgery: 82.35 % in the middle third, 9.81 % in the proximal third and 7.84 % in the distal third of the urethra (Table 2).

**Table 2:** Position of tapes

Position of the tapes	at 3 months after surgery	at 6 months after surgery	at one year after surgery
Proximal third of the urethra	3,92 %	9,81 %	9,81 %
The middle third of the urethra	88,23 %	82,35 %	82,35 %
Distal third	7,84 %	7,84 %	7,84 %

## Discussion

Our results show that the transversal incision of the vaginal mucosa favors the remaining of the sling in the mid-urethral area. We consider that the reason for our better percentage is represented by the limited dissection along the urethra.

The standard technique starts with a midline incision of the vaginal mucosa between two Allis clamps, the first one placed 1 cm proximal to the external urethral meatus and the second one 2–3 cm cephalad [7]. This allows the dissection of the peri-urethral space in the area of the mid-urethra (Fig. 1b). The longitudinal incision creates more space where the vagina is detached from the pre-urethral tissue, compared to the transversal incision. This may offer some space for the tension free tape to slide towards the bladder neck or proximal urethra. Our technique creates a space of maximum 1.5 cm along the long axis of the urethra, where the vagina is detached from the urethra. This way, the 1 cm width tape has little space to slide cranially or caudally (Fig. 2b). We consider that by performing a minimal dissection along the urethral axis, the tape is forced to remain at the level of mid-urethra, where it was initially placed.

Several studies investigated the location of the tape after TOT procedure using ultrasound. Jiang et al. 2013 assessed the position of the tape in group of 153 patients with previous TOT procedure. They found: 52.9 % of the tapes located in the proximal urethra, 30 % tapes in the middle urethra and 5.9 % in the distal urethra. Bogusiewicz et al. 2014 evaluated the position of the tape on a group of 141 patients and reported: 54 % of the tapes were located in the middle urethra, 36.17 % in the distal third of the urethra and 9.9 % in the proximal urethra [10, 11]. The results obtained with our technique were superior to those presented in literature, with 82.1 % of tapes located in the middle third of the urethra at 1 year after surgery.

It has been demonstrated [1] that the location of the tape is important in achieving continence, and that the middle third of the urethra is the optimal target for tape location [11]. Bogusiewicz et al. 2013 assessed a group of 61 patients with recurrent stress urinary incontinence following suburethral sling procedure and found that 73.8 % of the tapes were positioned at the level of proximal urethra, 4.9 % in the distal urethra and 21.3 % in the middle urethra [3]. Tape location in the proximal urethra or at the level of the bladder neck is considered a possible cause of de novo urgency and voiding dysfunction after TOT [4]. Yang et al. 2012 concluded that tape location in the proximal third of the urethra is responsible for the novo urgency and placement of the tape in the middle and distal third of the urethra is satisfactory [1, 11], while Lleberia et al. 2013 advocates that tape location outside the middle third increases the risk of the novo urgency and voiding difficulties [10]. Considering this, we believe that any maneuver that favors the remaining of the tape in the area of middle third urethra is valuable.

Our sample has 51 patients and the power of our study is over 90 %. We are aiming to extend our research to a greater number of cases and also to evaluate if we obtain better results in terms of lower incidence of de novo urgency and better continence rate using this type of incision. Using the transversal incision we create a suture line parallel to the sling which can lead to mesh exposure. We had no such cases but we need to validate that on a larger group of patients.

The main limitation of our study is represented by the lack of a control group including patients operated by the same team using the vertical incision. We considered the data available in literature for the vertical incision is very consistent hence we used it for comparison.

## Conclusion

In conclusion we strongly believe that this type of incision offers the advantage of a minimal dissection along the axis of the urethra and by this reduces the possibility of tape movement outside the middle third urethral zone.

## Abbreviation

TOT: Transobturator tape

## References

[1] Yang JM, Yang SH, Huang WC, Tzeng CR (2012). Correlation of tape location and tension with surgical outcome after transobturator suburethral tape procedures. Ultrasound Obstet Gynecol.

[2] Yang JM, Yang SH, Huang WC (2008). Dynamic interaction involved in the tension-free vaginal tape obturator procedure. J Urol.

[3] Bogusiewicz M, Monist M, Stankiewicz A, Woźniak M, Wieczorek AP, Rechberger T (2013). Most of the patients with suburethral sling failure have tapes located outside the highpressure zone of the urethra. Ginekol Pol.

[4] Jiang Y-H, Wang C-C, Chuang F-C, Ke Q-S, Kuo H-C (2013). Positioning of a suburethral sling at the bladder neck is associated with a higher recurrence rate of stress urinary incontinence. J Ultrasound Med.

[5] Staack A, Vitale J, Ragavendra N, Rodríguez LV (2014). Translabial ultrasonography for evaluation of synthetic mesh in the vagina. Urology.

[6] Delorme E, Droupy S, de Tayrac R, Delmas V (2004). Transobturator tape (Uratape): a new minimally-invasive procedure to treat female urinary incontinence. Eur Urol.

[7] Hoffman BL, Schorge JO, Schaffer JI, Halvorson LM, Bradshaw KD, Cunningham FG (2008). Williams gynecology, the McGraw-Hill Companies.

[8] Petros PE (2013). The female pelvic floor–function, dysfunction and management according to the intergral theory.

[9] Chow SC, Shao J, Wang H (2007). Sample size calculation in clinical research.

[10] Lleberia J, Pubill J, Mestre M, Aguiló O, Serra L, Canet Y (2013). De novo urgency: a review of the literature. Gynecol Obstet.

[11] Bogusiewicz M, Monist M, Gałczyński K, Woźniak M, Wieczorek AP, Rechberger T (2013). Both the middle and distal sections of the urethra may be regarded as optimal targets for ‘outside-in’ transobturator tape placement. World J. Urol. 2014; Feb 17. [Epub ahead of print]. J Ultrasound Med.

